# Food Prices

**Published:** 1920-01-31

**Authors:** 


### THE PUBLIC WELL-BEING.

### FOOD PRICES.

### Public Demand for Protection.

The provisional announcement of the Food Controller that food control will end in August perhaps ought not to be taken too seriously. Noah sent out the dove from the ark on an experimental mission, and it had to be repeated before Noah obtained the knowledge for which he sought. But it looks fairly certain that the Food Controller's dove will return—even if it has not already done so—with a somewhat aggressive kind of twig from its very first sally. The public is in no complacent temper just now on the subject of prices in food, as in everything else. Economic laws have made a certain increase inevitable, but when the public finds the official *Labour Gazette* showing an increase of 136 per cent. in the cost of food, concurrently with the disclosure of exorbitant profits and increased prices of many necessaries of life, it may be forgiven for being, to say the least, suspicious.

There has been a loud and insistent demand in certain quarters for the removal of Government control, and many specious reasons have been given. The average man may not be well versed in the details of cause and effect, and no doubt may be perfectly ready to accept the proposition that, as a general rule, the less the State has to do with commerce and trade the better. But being a simple man living by experience he has found that, whenever any given article is decontrolled, it rushes up in price, or that, when control is temporarily exercised, the article mysteriously disappears altogether until, by some stroke of magic, it leaves its lair when it hears the gladsome tocsin of a higher control price. The average man has to pay all the time. When the shackles slipped off margarine up went the price, and the Food Ministry had to come on the scenes again. It was the same with bacon and with veal. Potatoes and rice also did the Jack-in-the-box performance when the lid was released, and as for the case of wool no man can trust himself to speak of it temperately. We are now promised a reduction in the price of milk when control is removed. We shall have to follow the much-distorted spirit of the advice to "wait and see."

It is a matter of serious concern to consumers; their organisation, together with labour organisation, is already showing strenuous opposition to the suggested demobilisation of the Food Ministry. The Ministry itself has already warned the country that the position in the winter and spring of 1920-21 is uncertain, and the Parliamentary Secretary of the Ministry has said 1920 will be the most critical year since the war, at any rate as regards supplies and prices of butter and cheese. Yet in these most critical periods, officially designated, the public is threatened with a step which will leave it powerless in the hands of those who are ready to exploit them and the health of the community in general.

The cost of the prime necessities of life is already an extremely acute problem for all hospitals and such-like institutions, and they have a right to demand reasonable protection. Take only the supply of milk. It is dinned into the ears week in and week out that an adequate supply of good pure milk at reasonable prices is a vital factor in health, especially that of the young. It is no use preaching that doctrine to those who cannot afford the price now or about to be asked, and every time suggestions are made for the improvement so urgently needed in the quality, cleanliness, and handling of milk, they are met with the retort that it would cost too much. Everyone with any knowledge of farms and milk transport knows that the general conditions are altogether unsatisfactory, and whatever the difficulties of reform may really be, they ought to be overcome. It is futile to continue in the bad old ways, and the alternative to an efficient and economic remodelling of methods is a demand to force the hands of the milk industry by means of the Milk Act, municipalisation of milk, and so forth. But it would be better for the industry to pull itself together and take the necessary steps without continuous plaints of hard times. It has never been more prosperous than now.

There is no doubt the Food Ministry has done much in the interests of the public, and it ought to be retained until we return to more normal times.

